# Ocean acidification causes fundamental changes in the cellular metabolism of the Arctic copepod *Calanus glacialis* as detected by metabolomic analysis

**DOI:** 10.1038/s41598-022-26480-9

**Published:** 2022-12-23

**Authors:** Peter Thor, Fanny Vermandele, Allison Bailey, Ella Guscelli, Léa Loubet-Sartrou, Sam Dupont, Piero Calosi

**Affiliations:** 1 Norwegian Polar Institute, Fram Centre, 9296 tromsö, Norway; 2 Département de Biologie, Chimie et Géographie, Université du Québec à Rimouski, 300 Allée des Ursulines, Rimouski, QC G5L 3A1 Canada; 3 Department of Biological and Environmental Sciences, University of Gothenburg, 566 Kristineberg, 45178 Fiskebäckskil, Sweden; 4 Marine Environment Laboratories, International Atomic Energy Agency, 98000 Monaco, Principality of Monaco; 5 Present Address: Department of Aquatic Resources, Institute of Marine Research, Swedish University of Agricultural Sciences, Turistgatan 5, 45330 Lysekil, Sweden

**Keywords:** Climate-change ecology, Marine biology, Metabolomics

## Abstract

Using a targeted metabolomic approach we investigated the effects of low seawater pH on energy metabolism in two late copepodite stages (CIV and CV) of the keystone Arctic copepod species *Calanus glacialis*. Exposure to decreasing seawater pH (from 8.0 to 7.0) caused increased ATP, ADP and NAD^+^ and decreased AMP concentrations in stage CIV, and increased ATP and phospho-L-arginine and decreased AMP concentrations in stage CV. Metabolic pathway enrichment analysis showed enrichment of the TCA cycle and a range of amino acid metabolic pathways in both stages. Concentrations of lactate, malate, fumarate and alpha-ketoglutarate (all involved in the TCA cycle) increased in stage CIV, whereas only alpha-ketoglutarate increased in stage CV. Based on the pattern of concentration changes in glucose, pyruvate, TCA cycle metabolites, and free amino acids, we hypothesise that ocean acidification will lead to a shift in energy production from carbohydrate metabolism in the glycolysis toward amino acid metabolism in the TCA cycle and oxidative phosphorylation in stage CIV. In stage CV, concentrations of most of the analysed free fatty acids increased, suggesting in particular that ocean acidification increases the metabolism of stored wax esters in this stage. Moreover, aminoacyl-tRNA biosynthesis was enriched in both stages indicating increased enzyme production to handle low pH stress.

## Introduction

Ocean acidification (OA) is transforming the chemistry of the global ocean. The increased atmospheric CO_2_ partial pressure is pushing CO_2_ into the oceans where it reacts with water to form bicarbonate ions and H^+^, ultimately leading to a reduction in seawater carbonate ions and pH, and increase in bicarbonate ions. In the Arctic, the decrease in seawater pH is worsened by a concurrent decrease in the buffering capacity of surface waters due to increased sea ice melt and increased Atlantic water inflow carrying large amounts of anthropogenic CO_2_ to the Arctic Ocean^1,2^. On an inter-annual timescale, Arctic organisms are therefore exposed to much faster changes in the carbonate system than marine organisms from any other region. To add insult to injury, the physiological adaptations characterising true Arctic organisms may exacerbate the effect of these changes. Contrary to lower latitude eurytherms, polar species have low energetic costs for physiological maintenance at low temperatures^3^. While this enables high growth rates, lower energy expenditure also entails lower capacity for physiological regulation for homeostasis, including acid–base regulation^4^. Consequently, true Arctic species may be less capable of regulating metabolic effects of OA when compared to species with higher energy budgets^3^. Nevertheless, studies on OA effects in Arctic species are scarce, even for those species central to the functioning of Arctic marine ecosystem.

*Calanus glacialis* Jaschnow, 1955 is a keystone species in the Arctic shelf seas^5^. The effects of climate change and OA on this species are therefore central to understanding the future Arctic marine ecosystem. *Calanus glacialis* dominate the Arctic mesozooplankton biomass, and form a pivotal trophic node between the microplankton they feed upon and a wide range of predators such as teleost fish, baleen whales, and marine birds^6–8^. As an Arctic species, *C. glacialis* is adapted to the low-maintenance life of polar species^9^. Moreover, the strong seasonality of the Arctic has moulded its ontogeny to include migration to the deep for hibernation during the low production winter period. Therefore, while development during the main part of their nauplii and copepodite larval life is directed towards somatic growth and development, during stage CV, *C. glacialis* enters a different metabolic realm in preparation for diapause. They amass fatty acids and stores them in the form of wax esters (i.e. an ester of a fatty acid and a fatty alcohol), often constituting up to 80% of their body mass, and fatty acids from these stores are then used for egg production after maturation when exiting hibernation during the next Arctic spring^10^.

Although some studies show that some copepod species may be tolerant to OA by, for instance, increasing their food intake^11,12^, other studies have shown detrimental effects in the larval and juvenile stages of *C. glacialis*. While egg hatching seems unaffected^13^, Lewis and colleagues^14^ reported increased mortality at pH_T_ 7.8 (total scale pH) of nauplii larvae caught from under the ice in the high Canadian Arctic. Other studies show that surviving nauplii of *C. glacialis* grow and develop normally at OA levels down to pH_T_ 7.47 but only after alteration of fundamental physiological functions facilitated by changes in the expression of genes coding for DNA repair and transcription^15,16^. In the copepodites, effects vary with developmental stage. While stage CIII experiences changes in the metabolic response to feeding, stage CV copepodites and adults show no changes in ingestion rate, metabolic rate, gonad maturation rate or mortality^17–19^.

Our recent work shows significant decrease in ingestion rate under OA with a concomitant increase in metabolic rate in copepodite stage CIV, and further confirmed the results of Hildebrandt and colleagues that these traits do not change in stage CV^17,18,20^. In stage CIV, one week exposure to low pH caused a decrease in ingestion rate concomitant with an increase in metabolic rate^20^. This resulted in a significantly altered metabolic balance, with 19–50% reductions in scope for growth, potentially leading to reduced growth and development, and thus ultimately impaired fitness.

With the study presented here, we delved deeper into the biochemical underpinnings of the metabolic changes reported for *C. glacialis* stages CIV and CV by Thor et al.^20^. Using samples of copepodites from this work, we employed a targeted metabolomic approach to analyse how exposure to decreasing seawater pH affects the energy metabolism of *C. glacialis* and which molecular substrates are catabolized to supply this energy. *Calanus* copepods are predominately herbivorous and feeding individuals rely largely on phytoplankton hydrocarbons in the diet for energy production, but during periods of food scarcity copepods can revert to protein or lipid catabolism^21^. We therefore focused the analysis on 63 key molecules involved in the metabolism of hydrocarbons, proteins and lipids namely glucose, amino acids, fatty acids, and intermediates of the tricarboxylic (TCA) cycle, to shed light on the potential metabolic changes occurring in stage CIV and CV *C. glacialis* along a discreet seawater pH gradient.

## Methods

### Copepod exposure

Here we provide a brief description of the field collection of copepods, and the experimental design and procedure employed. Please refer to Thor et al.^20^ for further details.

Copepods were caught by vertical tows of a 200 µm WP2 net equipped with a closed cod end from 100 m to the surface in the Kongsfjord, Svalbard (79.0° N, 11.7° E) during July 2015 and transported to a temperature-controlled room (set at 5 °C) at the nearby Kings Bay Marine Laboratory (Ny-Ålesund, Svalbard). *Calanus glacialis* copepodites, stages CIV and CV, were selected under the stereomicroscope. They were discriminated from *Calanus hyperboreus* on the basis of prosome size, and lack of lateral spikes on the distal prosome segment and from *C. finmarchicus* by red pigmentation on the antennules^22–24^. While red pigmentation reliably distinguished *C. glacialis* from *C. finmarchicus* in live females from Disko Bay^22^, a later study demonstrated that the method was not 100% diagnostic in discriminating the two species at other sites, or for other developmental stages^25^. However, the error rate varies by site, and based on Choquet and colleagues’ data from Svalbard^25^, the proportion of *C. finmarchicus* copepodites in a selection of red-pigmented stage CIV and CV copepodites is likely low (< 2%).

CIV or CV copepodites were incubated in 28 glass bottles (620 mL), ten copepodites *per* bottle. Each bottle was filled with seawater adjusted to one of the 7 targeted pH levels, four replicates *per* pH level. Bottles were placed on a slowly rotating plankton wheel (0.5 rpm) at ca. 5 °C in dim light, for a total of 8 d. Every day approximately 500 mL of water in each bottle was renewed with freshly prepared water at target pH and food concentration.

Incubation water was prepared by mixing 0.3 µm filtered seawater (*fsw*) with small volumes of *fsw* acidified to ca. pH 5.5 by CO_2_ bubbling (Mapcon© CO_2_, Yara Praxair, Tromsø, Norway). Targeted pH on the total scale (pH_T_) levels were: 8.1, 7.9, 7.7, 7.5, 7.3, 7.1 and 6.9 (please refer to Thor et al*.*^20^ for measured values). Total alkalinity (A_T_) was analysed by potentiometric titration and total dissolved inorganic carbon (C_T_) was analysed by coulometric titration (Dickson et al*.*, 2007). *p*CO_2_ and pH_T_ were calculated using CO2SYS^26^. pH and electric potential (mV) were measured during the preparation of the batch incubation water using an electrode (InLab 413 SG/2 m, Mettler-Toledo, Columbus, OH, USA) connected to a calibrated pH meter (SevenGo SG2, Mettler-Toledo). Subsequent determination of pH_T_ from these measurements was accomplished using a standard curve established from simultaneous measurements in water samples of electric potential with the pH electrode and determination of pH_T_ from A_T_ and C_T_. For food, a paste of the diatom *Thalassiosira weissflogii* (Tw 1200, Reed Mariculture, Campbell, CA, USA) was added to a final concentration of ca. 10 µg Chl *a* L^−1^.

### Collection of copepods for metabolomic analysis

Copepods were collected at the end of the 8-d incubation period by pouring the contents of each bottle into a 20 µm mesh sieve held under water. Copepods in the sieve were then gently flushed into a Petri dish and examined under a stereomicroscope to determine developmental stage. From each bottle, one copepod was collected using a plastic pipette, dabbed on a laboratory tissue to remove excess water, placed in a dry 1.5 mL Eppendorf tube and flash frozen in liquid nitrogen. Total handling time of live copepods prior to flash freezing was < 1 min. Samples were first stored at -80 °C, then shipped to the Marine Evolutionary and Ecological Physiology laboratory of the University of Quebec in Rimouski (UQAR—Rimouski, QC, Canada) in a dry shipper with liquid nitrogen and finally stored at -80 °C until metabolite analyses were carried out.

### Metabolomic analyses

A total of 63 key molecules involved in the metabolism of hydrocarbons, proteins and lipids namely glucose, amino acids, free fatty acids, and intermediates of the tricarboxylic (TCA) cycle were analysed. The characterisation of metabolomic profiles was carried out using the liquid chromatography-high resolution mass spectrometry (LC-HRMS) method by Lu et al*.*^27^ modified to small marine organisms as described by Thibault et al*.*^28^ and Noisette et al*.*^29^. Briefly, we used a fast “cold quenching, salt-eliminating” extraction using ammonium carbonate as an extraction solution in order to prevent the formation of salt adducts when injecting marine organism samples, which could have compromised the detection of ions. Targeted metabolites were extracted from the frozen individuals in the Eppendorf tubes. 250 μL of extraction solution (8:2 methanol:water–10 mM ammonium) were added to each tube and samples were crushed with a potter pestle (blue pre-sterilized, Axygen, Tewksbury, MA, USA). All samples were sonicated in an ultrasound bath for 3 s (model Symphony, VWR, West Chester, PA, USA) and centrifuged at 11,000 rpm for 3 min at 4 °C (5430R, Eppendorf, Hamburg, Germany). For each sample, 225 μL of the supernatant was transferred to an amber HPLC vial with insert (Wheaton, New Jersey, USA) and injected into a liquid chromatography system (Accela, Thermo Electron Corporation, San Jose, CA, USA) equipped with a 150 mm × 2 mm Luna C5 guard column for Phenomenex (Torrance, CA, USA), with condition of injection similar to those used by Thibault et al*.*^28^. For further details see Thibault et al.^28^.

### Data processing and statistical analyses

Analysis of the LC-HRMS data was accomplished using the software Xcalibur 2.0 (Thermo Fisher Scientific, Waltham, Massachusetts, USA) with a 10 ppm mass tolerance. Calibration curves were constructed using linear, linear log–log or quadratic log–log regressions using the area of the working standard solution by extract ion integration.

Missing values are common in data from LC-HRMS metabolomics analysis so results were initially processed through Metlmp 1.2^30^ using the “missing, not random” classification since missing values were caused by metabolite concentrations falling below the limit of quantification of the LC-HRMS analysis. In this way, metabolites with more than 30% missing values across the treatments were removed entirely, whilst missing values of the remainder metabolites were replaced using quantile regression imputation of left-censored data (QRILC)^30^. For normalisation, metabolites were divided into four categories: (1) molecules carrying energy or redox potential, (2) metabolites involved in glycolysis and the TCA cycle plus 8-oxo-2-deoxyguanosine, (3) amino acids and (4) fatty acids, and concentrations of each metabolite in each individual copepod were divided by the sum of concentrations of all molecules belonging to the same category. For the further analysis, all concentrations were finally auto-scaled (mean-centred and divided by the standard deviation of each variable) and, for some metabolites showing uneven variance along the pH gradient, *glog* transformed or square root transformed to stabilise variances using MetaboAnalyst 4.0^31^. The concentrations of most metabolites were *glog* transformed for the regressions but some were left untransformed and one was square-root-transformed. To test whether reduced pH affected metabolite concentrations, the relationships between normalized, auto-scaled, and transformed metabolite concentrations and pH were then tested by linear regressions following Shapiro-Wilks tests for normality.

Enriched metabolic pathways were identified by Metabolite Set Enrichment Analysis (MSEA) (MetaboAnalyst 4.0) using KEGG numbers of metabolites significantly affected by pH (identified with the linear regression analyses) for each copepodite stage. Subsequently, the same list of KEGG numbers was used for Metabolomic Pathway Analysis (MetPA) to detect enriched pathways with significantly altered concentrations of metabolites with high pathway impact (metabolites at important positions within the pathway and hence likely to have significant impact on the pathways). The *Drosophila melanogaster* metabolite library was chosen as reference, as it is the only arthropod library available.

## Results

Fourteen of the 63 molecules were removed from the analyses due to insufficient numbers of samples returning valid concentrations (e.g. due to concentrations under the detection limit). These were NADH, NADP, acetyl-coenzyme A, 8-hydroxyguanosine, 8-hydroxyguanine, hydroxyproline, NADPH, butyric acid, mead acid, docosadienoic acid, eicosadienoic acid, linolenic acid, and arachidonic acid.

Decreasing seawater pH significantly altered the concentrations of 16 and 13 of the remaining 49 metabolites, stage CIV and CV, respectively).

### Molecules carrying energy or redox potential

**Figure 1:**
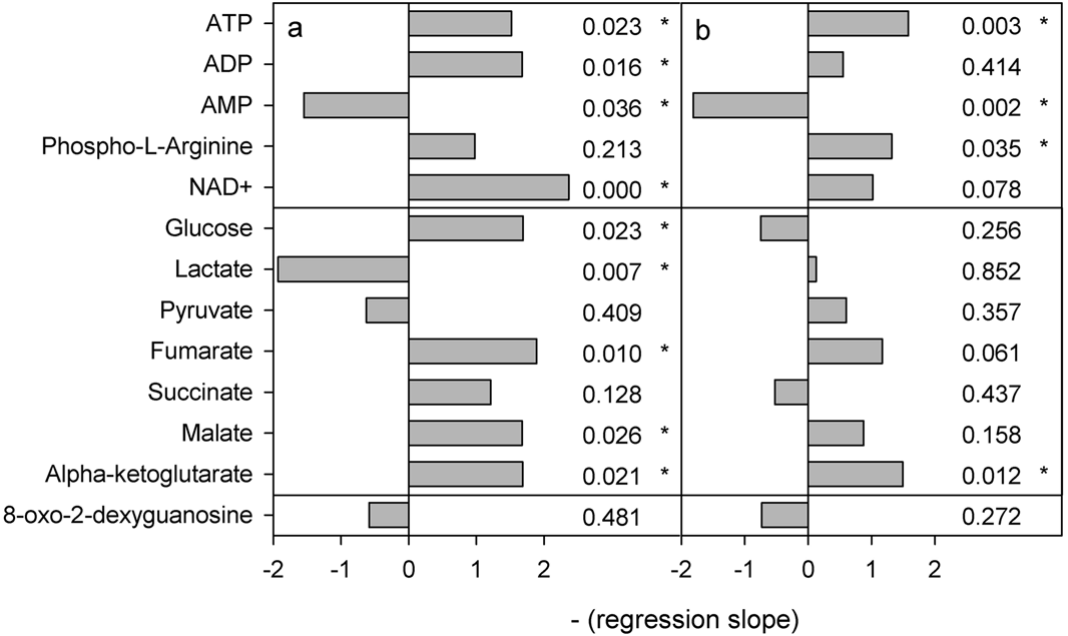
Results of linear regressions on transformed metabolite concentrations in *C. glacialis* copepodites as a function of pH (range 8.0–7.0 pH_T_). (**a**) Copepodite stage CIV, (**b**) copepodite stage CV. The horizontal grey bars show negative regression slopes to indicate the direction of change with decreasing pH and the numbers show the p-values of the slopes, asterisks mark significant slopes. Upper panels show molecules carrying energy or redox potential, middle panels show metabolites involved in glycolysis or TCA cycle and lower panel show a product of oxidative stress.

**Figure 2:**
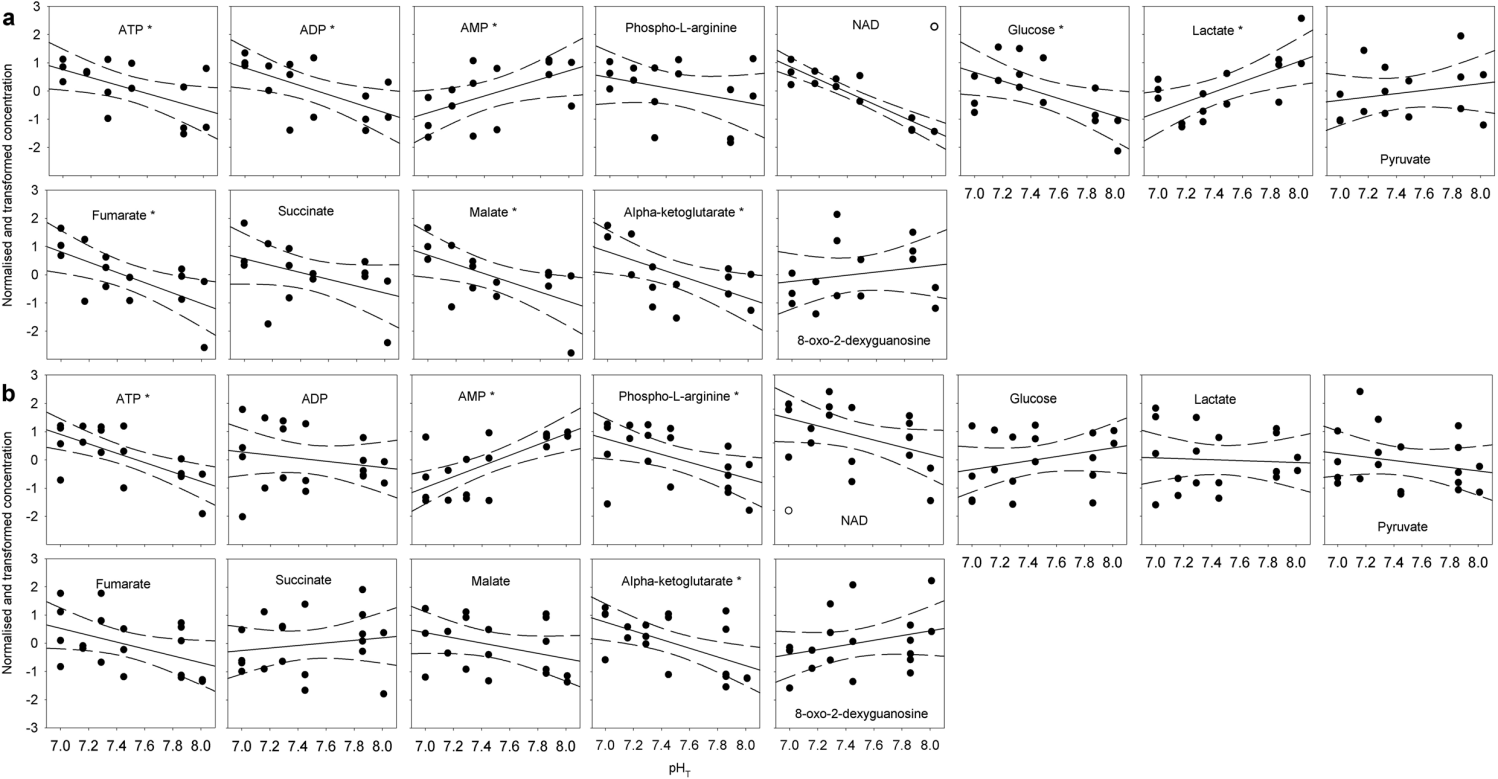
Normalised and transformed concentrations of molecules carrying energy or redox potential and metabolites involved in glycolysis and the TCA cycle. (**a**) Copepodite stage CIV, (**b**) copepodite stage CV. Lines show linear regressions and hatched lines show 95% confidence intervals. Point with white fill in NAD was distanced from the predicted y-value by more than double the 95% confidence interval and had considerable leverage on the regression. It was therefore excluded as an outlier. Asterisks in the molecule name denote significant slopes (P < 0.05).

The energy molecules ATP, ADP, and AMP showed a consistent pattern of change in response to pH in both developmental stages. ATP concentrations increased significantly with decreasing pH (linear regressions: CIV: *P* = 0.023, CV: *P* = 0.003) (Figs. 1, 2) along with increasing ADP concentrations (significant in stage CIV: *P* = 0.026). These increases were accompanied by significant decreases in AMP concentrations (CIV: *P* = 0.036, CV: *P* = 0.002). Phospho-L-arginine, also increased in both stages, significantly so in stage CV (*P* = 0.035). NAD^+^ increased in both stages, but only significantly in stage CIV (*P* < 0.001).

### Metabolites involved in glycolysis or TCA cycle

**Figure 3:**
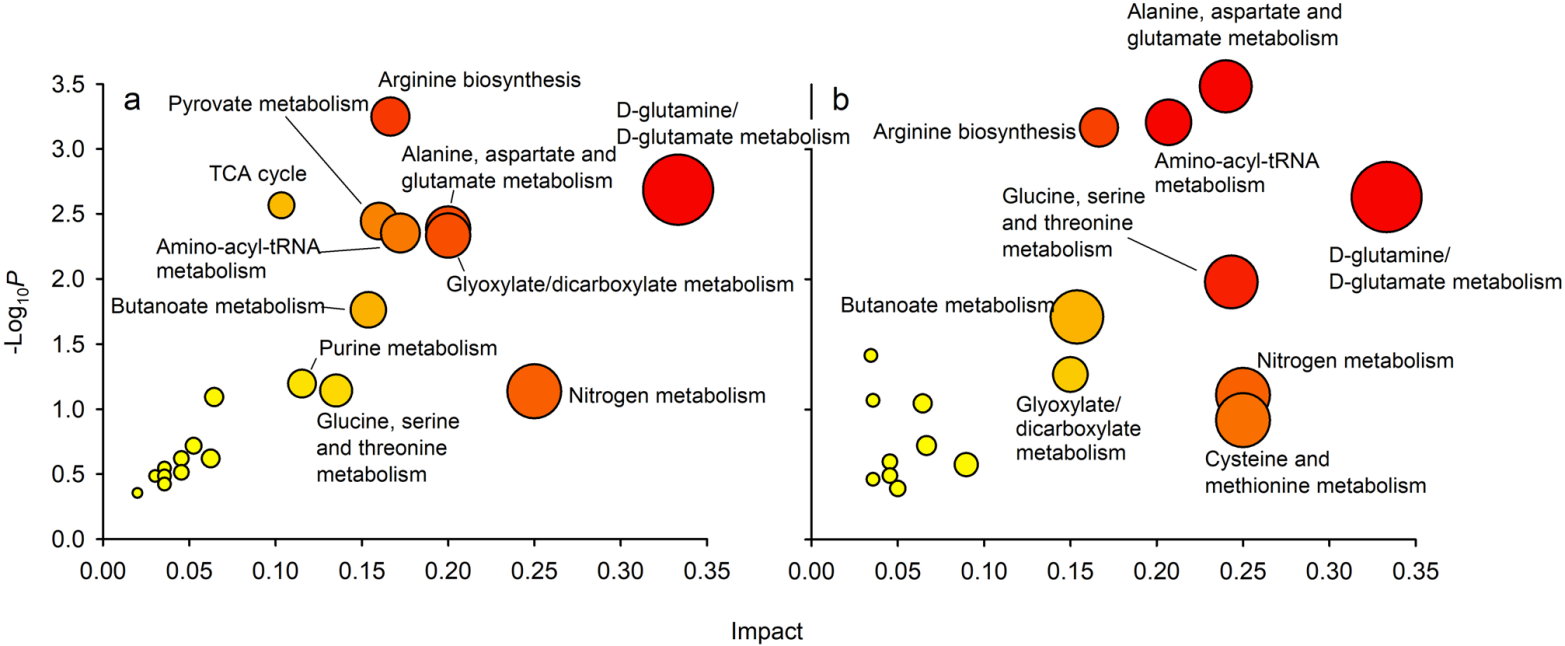
Metabolic pathway analysis (MetPA) results showing pathways affected by pH. (**a**) Copepodite stage CIV, (**b**) copepodite stage CV. The analyses were based on the hypergeometric test enrichment method and out-degree centrality pathway topology on the *Drosophila melanogaster* reference library. Y-axis show the significance of the test (*p*-value) and x-axis show the impact of the tested molecules on the pathway. Bubble size and colour indicate pathway impact size. Thus, pathways in the top right diagonal region contain significantly changed metabolites and, due to their important positions, these changes are more likely to have big impacts on the pathways.

Concentrations of metabolites involved in glycolysis and the TCA cycle were more often altered in stage CIV than in stage CV. In stage CIV, five of seven metabolites showed significantly changing concentrations with decreasing pH_T_ (Figs. 1, 2). Of these, glucose, fumarate, malate and alpha-ketoglutarate increased significantly (linear regressions: *P* < 0.05), whereas lactate decreased significantly (*P* = 0.007). Pathway analyses confirmed these findings for stage CIV, in which the TCA cycle was enriched (MetPA: impact 0.10, *P* = 0.0027), as was pyruvate metabolism (MetPA: impact = 0.16, *P* = 0.0036) (Fig. 3). The glyoxylate and dicarboxylate metabolism was also enriched (MetPA: impact = 0.20, *P* = 0.0047). In stage CV, five of the eight metabolites involved in glycolysis and the TCA cycle increased with decreasing pH, though only alpha-ketoglutarate increased significantly (*P* = 0.012). Despite non-significant correlations for the single metabolites, the TCA cycle was significantly enriched as a pathway (MetPA: impact = 0.15, *P* = 0.039) (Fig. 3).

### Amino acids

While both stage CIV and stage CV experienced significant changes in the concentrations of some free amino acids (six and five of the 14 amino acids investigated, stage CIV and CV, respectively), the direction differed by stage: in stage CIV they were both up- and down regulated, while stage CV copepodites they showed an overall down-regulation with decreased pH.

**Figure 4:**
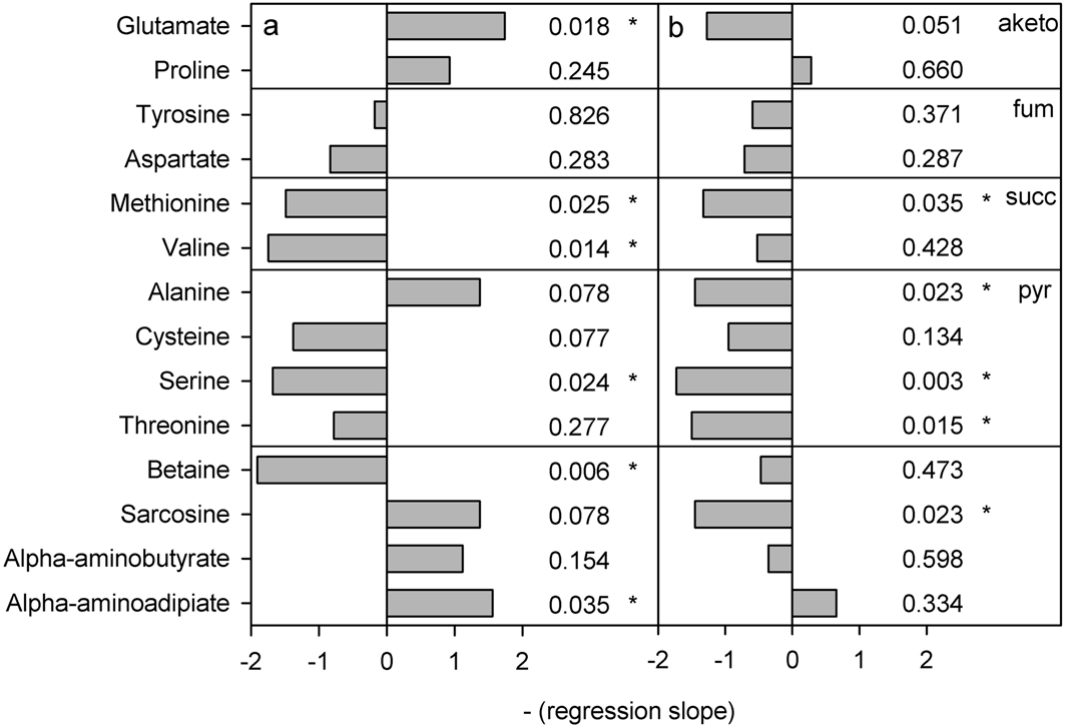
Results of linear regressions on transformed amino acid concentrations in *C. glacialis* copepodites as a function of pH (range 8.0-7.0 pHT). a) Copepodite stage CIV, b) copepodite stage CV. The horizontal grey bars show negative regression slopes to indicate the direction of change with decreasing pH and the numbers show the p-values of the slopes, asterisks mark significant slopes. Upper four panels show amino acids according to their entry point into the energy metabolism: from the top the four TCA cycle metabolites alpha-ketoglutarate, fumarate/oxaloacetate, and succinate followed by pyruvate in the glycolysis. The bottom four amino acids are not directly metabolised in the TCA cycle or the glycolysis.

**Figure 5:**
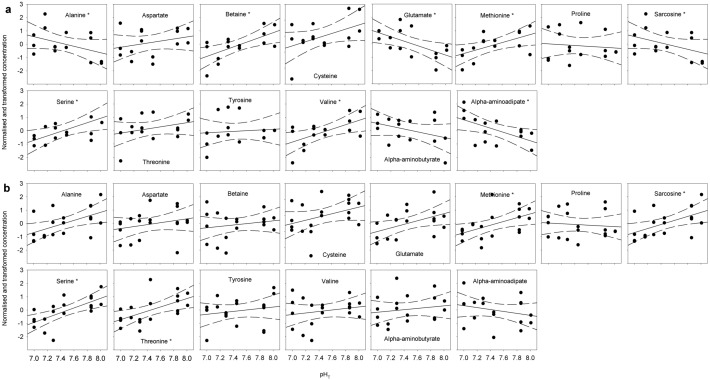
Normalised and transformed concentrations of amino acids. a) Copepodite stage CIV, b) copepodite stage CV. Lines show linear regressions and hatched lines show 95% confidence intervals. Point with white fill in NAD was distanced from the predicted y-value by more than double the 95% confidence interval and had considerable leverage on the regression. It was therefore excluded as an outlier. Asterisks in the molecule name denote significant slopes (P < 0.05).

Methionine and valine, which both enters the TCA cycle as succinyl-coA when catabolised, decreased in both developmental stages (all significant except valine in stage CV: *P* < 0.05) (Figs. 4 and 5). Also the amino acids catabolised to pyruvate, namely alanine, cysteine, serine and threonine, decreased in concentrations, except alanine in stage CIV (four of them significantly, *P* < 0.05). Glutamate increased significantly in stage CIV (*P* = 0.018). Betaine and alpha-aminoadipate, an intermediate in lysine synthesis, increased significantly in stage CIV (*P* = 0.035) and sarcosine decreased significantly in stage CV (*P* = 0.023).

A range of different amino acid metabolic pathways were enriched in the MetPA. Alanine, aspartate and glutamate metabolism pathways were significantly enriched in both developmental stages (MetPA, CIV: impact = 0.20, *P* = 0.0041, CV: 0.24, *P* = 0.0003) (Fig. 3). Moreover, glycine, serine and threonine metabolic pathways were significantly enriched in stage CV (MetPA: impact = 0.24, *P* = 0.011). Biosynthetic pathways were also enriched. Arginine biosynthesis was enriched in both stages (MetPA, CIV: impact = 0.17, *P* = 0.0006, CV: impact = 0.17, *P* = 0.0007), and so was synthesis of Aminoacyl-tRNA, a molecule taking part in protein synthesis (MetPA, CIV: impact = 0.17, *P* = 0.0045, CV: impact = 0.21, *P* = 0.0006). D-glutamine and D-glutamate metabolism was enriched in both developmental stages (MetPA, CIV: impact = 0.33, *P* = 0.002, CV: impact = 0.33, *P* = 0.002) and butanoate metabolism was significantly enriched in both developmental stages (MetPA: CIV: impact = 0.15, *P* = 0.017, CV: impact = 0.15, *P* = 0.020) but this pathway is exclusive to intestinal bacteria and was not considered further (Fig. 3).

### Fatty acids

**Figure 6:**
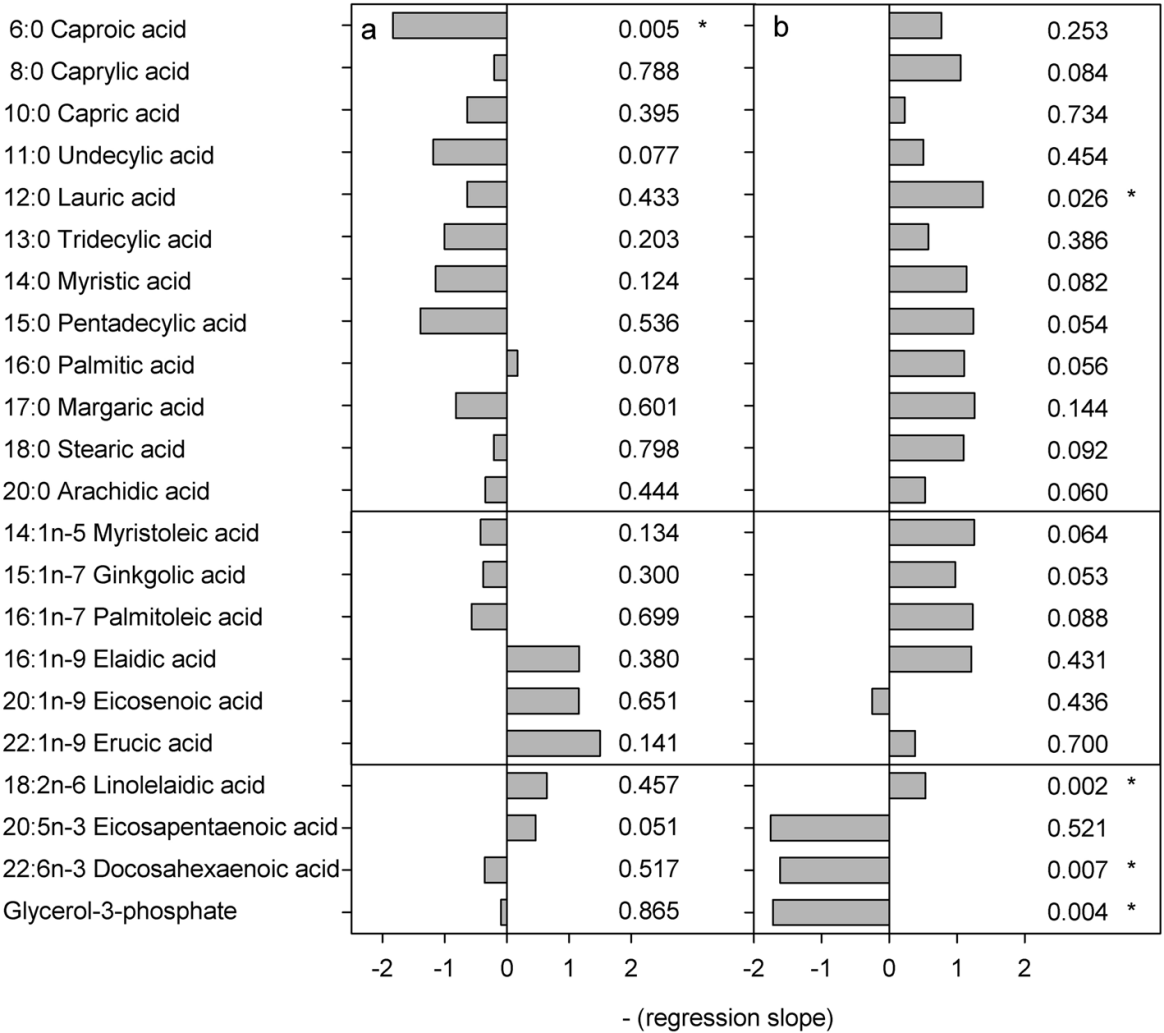
Results of linear regressions of transformed fatty acid concentrations in *C. glacialis* copepodites as a function of pH (range 8.0–7.0 pH_T_). (**a**) Copepodite stage CIV, (**b**) copepodite stage CV. The horizontal grey bars show negative regression slopes to indicate the direction of change with decreasing pH and the numbers show the p-values of the slopes, asterisks mark significant slopes. Upper panel shows saturated fatty acids, middle panel shows monounsaturated fatty acids and lower panel shows polyunsaturated fatty acids.

**Figure 7:**
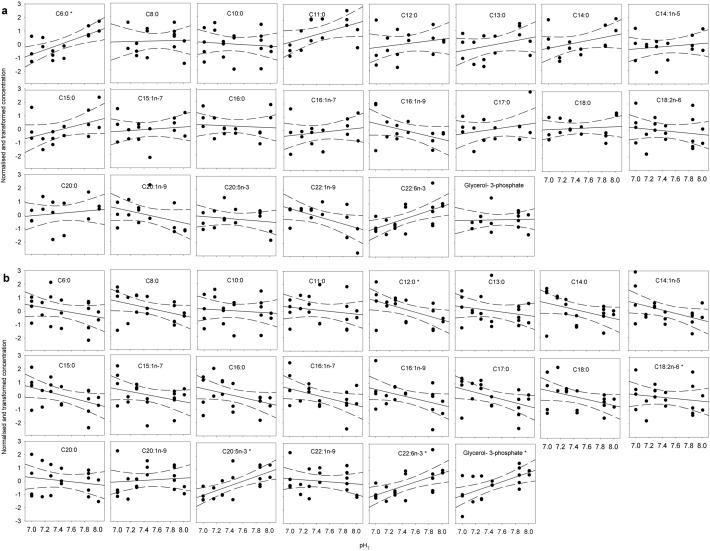
Normalised and transformed concentrations of fatty acids. (**a**) Copepodite stage CIV, (**b**) copepodite stage CV. Lines show linear regressions and hatched lines show 95% confidence intervals. Point with white fill in NAD was distanced from the predicted y-value by more than double the 95% confidence interval and had considerable leverage on the regression. It was therefore excluded as an outlier. Asterisks in the molecule name denote significant slopes (*P* < 0.05).

Generally, free fatty acid concentrations decreased with decreasing pH in stage CIV (16 of 21) and increased in stage CV (18 of 21), though few had significant slopes (1 and 3 of 21 for stage CIV and stage CV, respectively). For stage CIV, only C6:0 caproic acid decreased significantly (*P* = 0.005) (Figs. 6, 7). Of the many fatty acids that increased with decreased pH in CVs, only C12:0 lauric acid and 18:2n-6 linolelaidic acid increased significantly (*P* < 0.05). The two essential polyunsaturated fatty acid C20:3n-3 eicosapentaenoic acid and C22:6n-3 docosahexaenoic acid both decreased significantly in stage CV (*P* < 0.05). Moreover, glycerol-3-phosphate, a precursor of glycerophospholipids, a main component of cell membranes, decreased significantly in stage CV (*P* = 0.004) (Figs. 6, 7). No fatty acid pathways were identified as enriched in MetPA in either of the two developmental stages.

## Discussion

Using a targeted metabolomics approach, we showed that late copepodite stages of the keystone Arctic copepod *Calanus glacialis* experience important changes in several central energetic pathways following exposure to decreasing pH. These findings shed light on the physiological changes underpinning the effects of OA on fitness related traits such as ingestion rate and metabolic rate previously observed in this species^17,18,20^.

### Cellular energy metabolism

Cellular energy production was altered consistently in both stage CIV and CV, with concentrations of higher energy adenosine phosphates (ATP and ADP) increasing, and concentrations of the lower energy, less-phosphorylated AMP decreasing, with decreasing seawater pH. Moreover, Phospho-L-arginine, which in crustaceans functions as phosphagen in the replenishment of ATP from ADP during transient energy demands^32^, increased significantly in stage CV. These changes strongly suggest that exposure to low pH affects energy production and expenditure in both developmental stages, although with nuanced differences.

NAD^+^ increased significantly in stage CIV. NAD^+^ is an essential redox carrier receiving electrons from oxidative processes in the glycolysis, the TCA cycle, and fatty acid oxidation to form NADH. A high NAD^+^/NADH ratio facilitates higher rates of these reactions and thus potentially higher rates of ATP production (unfortunately, the LC-HRMS could not detect NADH). But most importantly, the produced NADH serves as electron donors to ATP synthesis in the oxidative phosphorylation. For every ATP produced in the oxidative phosphorylation one NADH is oxidised back to NAD^+^. High rates of ATP production in the oxidative phosphorylation would therefore amass NAD^+^, as observed in stage CIV. Conversely, ATP production in the glycolysis and TCA cycle consumes NAD^+^ (9 NAD^+^ *per* 4 ATP) and glycolytic ATP production would decrease the NAD^+^ concentration with decreasing pH.

Heterotrophic organisms generally face a trade-off between rate and yield of ATP production. The efficient low rate/high yield production in the TCA cycle/oxidative phosphorylation may prevail under certain circumstances, whereas under other circumstances, the less efficient high rate/low yield production in the glycolysis may predominate^33^. Because glycolysis and oxidative phosphorylation compete for ADP, the one dominate over the other in terms of rates depending on the substrate being metabolised. In stage CIV copepodites, the TCA cycle pathway was enriched in the MetPA, and metabolites associated with glycolysis and the TCA cycle showed significant changes in their concentrations at decreasing seawater pH. Glucose, the entry point to glycolysis, increased significantly with decreasing pH. High levels of blood glucose (hyperglycemia) have been observed as a general stress response in decapod crustaceans^34^. Copepods have no circulatory system (although they have a dorsal heart) but might nevertheless react similarly on the cellular level. Along with the significant increase in glucose, lactate decreased significantly with pH in stage CIV. Lactate is an inevitable end product of glycolysis, because lactate dehydrogenase has the highest *V*_max_ of any enzymes in the glycolytic pathway and the *K*_eq_ for pyruvate to lactate is far in the direction of lactate^35^. Accordingly, although the glycolysis was not enriched in the MetPA, conceivably because none of its intermediate metabolites were included in the analyses (the protocol did not allow for it), we hypothesise that stage CIV copepodites experience a general down-regulation of glycolysis under decreasing pH. Alternatively, the amassing of glucose and depletion of lactate could also indicate increased gluconeogenesis. Gluconeogenesis occurs during starvation to replenish glycogen stores and ingestion rates did decrease in stage CIV^20^. But again, we did not target any intermediates in our analyses, and thus cannot firmly conclude on this.

Phosphofructokinase-1 is a key regulatory enzyme of glycolysis^36^. This enzyme is allosterically inhibited by ATP and activated by AMP, and interestingly this regulation is augmented by low pH^37,38^. Thus, phosphofructokinase-1 could be key to the down-regulation of glycolysis we hypothesise. The fact that we found increasing oxygen consumption with decreasing pH in stage CIV copepodites from the same experiment adds further momentum to this line of thought^20^. It seems that stage CIV copepodites might experience the so-called *Pasteur effect*—a decrease in glycolysis at increased levels of oxygen uptake—when exposed to decreasing pH^39^. Although ATP and AMP were significantly affected also in stage CV, glucose, pyruvate and lactate did not change with decreasing pH, which perhaps indicate absence of the down-regulation of glycolysis we hypothesise for stage CIV. There is, nevertheless, one indication that down-regulation may in fact occur also in this developmental stage. Alpha-glycerophosphate decreased significantly with decreasing pH in stage CV. This molecule is an intermediate in the transfer of electrons from NADH produced by glycolysis in the cytosol to the oxidative phosphorylation in the mitochondria, and decreased concentrations could result from down-regulation of the glycolysis also in stage CV copepodites.

The TCA cycle was enriched for stage CIV and most of the measured TCA cycle metabolites (alpha-ketoglutarate, succinate, fumarate, and malate) showed increasing concentrations at decreasing pH. Trigg et al*.*^40^ observed a similar increase in concentrations of TCA cycle-related metabolites in the Dungeness crab, *Cancer magister* (Dana, 1852), at decreased pH and concluded that TCA cycle activity is upregulated under OA. Since NAD^+^ is the product of the transport of electrons from the TCA cycle to the oxidative phosphorylation in the mitochondria,  the increase in NAD^+^ concentration we observed in stage CIV could reflect an increase in the flow of electrons from the TCA cycle to the oxidative phosphorylation, and by extension an increase in the energy production by the TCA cycle and the oxidative phosphorylation. There is negative feedback from the TCA cycle to glycolysis through inhibition of phosphofructokinase-1 by citrate, a metabolite of the TCA cycle^38^. Unfortunately, we did not target citrate in our targeted approach to specifically test this hypothesis, but the amassing of NAD^+^ do provide additional support to the idea that glycolysis is down-regulated at decreasing pH. Again, there is a less clear picture of how cellular energy metabolism is affected by decreasing pH in stage CV when compared to stage CIV. There was no clear pattern of regulation of TCA metabolites, and the TCA cycle was not enriched in the MetPA. Nevertheless, alpha-ketoglutarate concentrations did increase with decreasing pH in CVs.

The glyoxylate/dicarboxylate cycle was also enriched in the pathway analysis, but this is probably also a result of the increases in concentrations of alpha-ketoglutarate, succinate, fumarate, and malate, and we are unable to distinguish it from the TCA cycle based on the set of metabolites analysed.

Conclusively, lowered glycolysis due to inhibition of phosphofructokinase-1 and upregulation of the TCA cycle and oxidative phosphorylation at low pH in stage CIV appear plausible causes for the changes in ATP, ADP and AMP concentrations we observed. Alongside these effects, down-regulation of transcription of genes involved in the glycolysis were also present in nauplii of *C. glacialis* exposed to 35–38 days of low pH conditions^16^. On the other hand, studies on the acclimatisation and adaptation to OA in another calanoid copepod species, *Pseudocalanus acuspes* (Giesbrecht, 1881), showed no increase in expression of mitochondrial genes at pH_T_ 7.54, which would have been expected if the TCA cycle or oxidative phosphorylation is upregulated^41^. Interestingly, De Wit et al*.*^41^ also showed natural selection in a large fraction of mitochondrial genes under OA conditions. Even evolutionarily conserved sequences, such as cytochrome oxidase subunit I, were under selection and it was hypothesised that the mitochondrial function of oxidative phosphorylation is a target for natural selection in copepods at low pH^41^.

Besides its role in the transfer of energy from the mitochondria to the cell, ATP is also used to fuel cell homeostasis and active cellular acid–base regulation by activation of ATP-dependent enzymes involved in osmo-ionic- and acid–base regulation. In crustaceans, acid–base status is linked to ion regulation, and is maintained primarily through ion transport mechanisms moving acid and/or base equivalents between the extracellular fluid and the ambient water^42^. One prominent process in this respect is regulation by Na^+^/K^+^-ATPase^42,43^. While this regulation takes place in the gills of decapod crustaceans^43^, it is located in the maxillary glands and other specialised organs on the swimming legs of copepods^44^. Any extensive ATPase mediated pH regulation could have manifested itself by decreasing ATP concentrations, but this is contrary to what we report here. Interestingly, while the *p*CO_2_-sensitive isopod *Cymodoce truncata* (Leach, 1814) is able to maintain its cellular ATP concentration at the expense of the concentration of carbonate anhydrase (an enzyme involved in the cellular transformation of water and CO_2_ to bicarbonate ions and H^+^ prior to the ATPase mediated transport of H^+^ across the cell membrane), the *p*CO_2_-tolerant isopod *Dynamene bifida* (Torelli, 1930) upregulates ATP with no functional compromise to CA concentrations^45^. Finally, *C. glacialis* nauplii have shown upregulation of Na^+^/H^+^-antiporters independent of ATPase as a response to OA^16^, which one could hypothesise also may be the case in the copepodites. Arctic populations of the amphipod *Gammarus setosus* also do not experience increased ATPase activity during OA conditions^46^. It seems that *C. glacialis* faces OA without any ATP dependent acid/base regulation activity.

Glycolysis is the first step of catabolism of carbohydrates for the production of energy. When down-regulating glycolysis the copepods may be increasingly dedicated to catabolism of amino acids e.g. through oxidative deamination of glutamate and/or catabolism of fatty acids through beta-oxidation to produce the energy they require^21^. Both lead to the production of molecules entering the TCA cycle and ultimately the oxidative phosphorylation for energy production in the mitochondria.

### Amino acid metabolism

Of the free amino acids which were significantly affected by decreasing pH, the majority decreased in concentration, for both stage CIV and CV copepodites. This could be an indication of changes in protein synthesis at decreasing pH. Supporting this idea, biosynthesis of aminoacyl-tRNA was indicated as significantly enriched in the MetPA in both stage CIV and CV. Aminoacyl-tRNA partakes in the elongation of the protein amino acid chain during protein synthesis and the enrichment was most likely due to the changes in concentration of the many amino acids tested. One probable cause of protein synthesis is the increased demands of enzymes needed to handle stress at low pH, including for example enzymes involved in acid–base- and osmo-regulation or regulation of energy production. Increased protein synthesis caused by OA conditions has been observed in larvae of the purple sea urchin *Strongylocentrotus purpuratus* (O.F. Müller, 1776)*,* where in vivo rates of protein synthesis and ion transport increased ∼50%^47^. Costs of protein synthesis are high and have shown to constitute a major part of copepod metabolic demand^48^ and we did observe significant increases in metabolic rate in copepodite stage CIV from the same experiment^20^ giving further credit to the idea that protein synthesis was upregulated.

An alternate but not mutually exclusive explanation is that the copepods experience increased amino acid catabolism under OA. Glutamate increased in stage CIV accompanied by a significant increase in alpha-ketoglutarate in both stage CIV and CV. Alpha-ketoglutarate is part of the metabolic pathway of glutamine, glutamate and arginine in which glutamate acts as an intermediate in catabolism of these amino acids when it is deaminated to alpha-ketoglutarate to enter the TCA cycle^49^. Glutamate metabolism (in conjunction with alanine and aspartate metabolism) was significantly enriched in the MetPA in both stage CIV and CV, and these changes could be taken as an indication of a shift towards amino acid catabolism with decreasing pH. The key enzyme catalysing the oxidative deamination of glutamate is glutamate dehydrogenase (GDH), which functions in both directions: deamination of glutamate to form alpha-ketoglutarate or formation of glutamate from alpha-ketoglutarate. Studies on the ribbed mussel, *Modiolus dernissus* (Dillwyn, 1817), have shown that the balance of this action is strongly pushed towards deamination when pH decreases from 8.0 to 7.5^50^. GDH is activated by ADP, and one could argue that the increase in ADP we observed would work against this shift, but ADP activates GDH mainly in the glutamate forming direction^51^. The other measured amino acids enter the TCA cycle at different positions we unfortunately could not target in our analyses. Glutamate also partakes in the arginine biosynthesis pathway in which it is transformed to ornithine to enter the urea cycle. Arginine biosynthesis was enriched in the MetPA and it is therefore possible that decreasing pH also changes amino acid catabolism to increase urea excretion. Decreasing pH has a similar depressing effect on amino acid concentration in the gills of the shore crab *Carcinus maenas* (Linnaeus, 1758) which also has been interpreted as a sign of increased protein catabolism^52^. Hammer and colleagues^52^ argued that this increase in catabolism served to buffer H^+^ by supplying nitrogen to NH_4_ formation in the cells. All in all, we hypothesise that increased amino acid catabolism, possibly driven by changes in GDH activity, and the down-regulation of glycolysis by inhibition of phosphofructokinase-1 may be major drivers of a shift from carbohydrate metabolism towards catabolism of amino acids.

D-glutamine/D-glutamate metabolism was highly enriched in the MetPA in both developmental stages. Several studies show enriched D-glutamine/D-glutamate metabolism in crustaceans [e.g. 53], but they offer no explanation of its function or the reason why it is enriched. While D-glutamate act in neurotransmission, this action is evolutionarily restricted to ctenophores, and biochemical measurements of D-amino acid concentrations have shown absence of D-glutamate in crustaceans^54,55^.

We observed no changes in concentrations of 8-oxy-2-deoxyguanosine, a product of DNA oxidation. Furthermore, regulation of cellular response to oxidative stress is down-regulated in *C. glacialis* nauplii^16^, and OA may not induce oxidative stress in *C. glacialis*.

### Fatty acid metabolism

Besides their importance in energy storage as wax esters, fatty acids are involved in many central processes in cells, most prominently through their function as cell membrane building blocks. Many fatty acids are obtained from the diet but some longer chain fatty acids, such as 20:1n-9 are synthesised de novo in copepods^56^. Stage CV copepodites experienced increases in most of the targeted free fatty acids (18 of 21) with decreasing pH. Only one of those 18 increased significantly, but since the direction of change were the same in all, we argue that the pattern of change does merit consideration. Conspicuous exceptions were eicosapentaenoic acid (EPA) 20:5n-3 and docosahexaenoic acid (DHA) 22:6n-3, which both decreased significantly. The only other study (to our knowledge) of metabolomic effects of environmental changes in copepods showed the exact same response to starvation in a mix of *C. finmarchicus* and C*. helgolandicus* stage CV copepodites, with most fatty acids increasing while EPA and DHA decreased in concentration^57^. EPA and DHA are key marine polyunsaturated fatty acids (PUFAs) exclusively produced by marine algae. They contribute a major fraction of the fatty acids of cell membrane phospholipids^58^, and zooplankton reproductive production is highly dependent on especially EPA^59^. EPA and DHA are key for cell membrane fluidity, which for calanoid copepods is especially important during diapause in the deep during copepodite stage CV^60^. They have also been linked to diapause buoyancy control, and are selectively metabolized in diapausing copepodites^61^. The importance of EPA and DHA for cell membrane integrity may be central for the changes we observed. Glycerol-3-phosphate, the precursor for the glycerol backbone of cell membrane phospholipids also decreased significantly and it seems decreasing pH could affect cell membrane turnover.

Changing fatty acid concentration could be due to either a change in lipid intake from feeding or increased fatty acid catabolism. While ingestion rates decreased in stage CIV, they were unchanged in stage CV with decreasing pH^20^. Also, *Thalassiosira weissflogii *(Grunow) G.Fryxell & Hasle, 1977, the diatom we fed to the copepods, is rich in 16:0, 16:1n-7 and EPA^59^. The concentrations of 16:0 and 16:1n-7 increased, whereas EPA concentration decreased. If fatty acid concentrations reflected feeding, we would have seen increased concentrations of all three. We therefore believe that the general increases in concentrations of free fatty acids were caused by increasing catabolism of the wax esters stored in stage CV. It may be that due to the metabolic reconfiguration to enter hibernation, stage CV copepodites are already committed to the catabolism of fatty acids through beta-oxidation, and stored wax esters are being hydrolysed to increase the availability of free fatty acids for energy production. Mayor and colleagues^57^ arrived at the same conclusion. We hypothesise that stress due to low pH increases the organism’s energetic demands, but carbohydrates are not used to accommodate these demands due to the down-regulation of the glycolysis, rather demands are met by hydrolysing and metabolising wax esters in stage CV. The further ramifications of future OA could therefore be a less efficient build-up of wax esters so important for hibernation in this species.

Finally, besides their importance for cell membrane fluidity, EPA and DHA are important precursors for eicosanoid endocrine hormones. These hormones are important regulators of, among other processes, ion flux^62^. As mentioned above, acid base regulation is coupled to osmoregulation in crustaceans^42^, and the decrease in concentrations of these two specific fatty acids, when all other fatty acid concentrations increased might represent an indication for changing endocrine hormone production to counter adverse whole-organism effects of OA.

Changes in metabolite concentrations cannot be directly translated into changes in the rate of the processes they are involved in. However, they do pin-point processes which are affected by the imposed environmental changes. Also, in our analyses we targeted a limited range of molecules. In that respect OA could inflict changes in other important metabolic pathways we did not investigate. The absence of specific biochemical pathways in our analyses and discussion should therefore not be taken as indication that these are not implicated in this species responses to OA.

From our previously published study on copepodites from the same incubations, we know that high *p*CO_2_/low pH conditions have detrimental effects on the balance between energy input (ingestion) and energy expenditure (metabolism) in stage CIV copepodites but not in stage CV copepodites^20^. The effects we report here help in this sense to shed light on the metabolic origin of the rather severe effects on energy balance we observed in stage CIV copepodites and the difference in response between stage CIV and CV^20^. Copepods develop through six nauplii and five copepodite stages before maturation, and while previous studies show negligible effects in stage CV and adults^17,18,20^, any effects in any developmental stage along the way will affect the fitness of the individual and the recruitment to the population as a whole. In addition, the enhanced fatty acid metabolism observed in stage CV needs further investigation, to determine the magnitude of the fitness implications of the energy diverted away from energy storage for hibernation.

## Supplementary Information

Supplementary Information.

## Data Availability

All data are available in the supplementary material.
